# HADH may be the target molecule of early vascular endothelial impairment in T2DM

**DOI:** 10.3389/fcvm.2022.963916

**Published:** 2022-08-10

**Authors:** Haowen Ye, Ruxin Wang, Jinjing Wei, Ying Wang, Lihong Wang, Xiaofang Zhang

**Affiliations:** ^1^Department of Endocrinology and Metabolism, First Affiliated Hospital of Jinan University, Guangzhou, China; ^2^Department Clinical Experimental Center, First Affiliated Hospital of Jinan University, Guangzhou, China

**Keywords:** WGCNA, GEO, T2DM, AS, endothelial cell dysfunction

## Abstract

**Background:**

Type 2 diabetes mellitus (T2DM) will significantly increase the risk of atherosclerosis (AS). Vascular endothelial cell dysfunction (VECD) is the foundation of AS. Early identification and intervention of VECD caused by T2DM can help us effectively delay or even suppress the occurrence of AS.

**Methods:**

We downloaded the gene expression profiles from the Gene Expression Omnibus (GEO). The differential expression genes (DEGs) were identified in R software and weighted gene co-expression network analysis (WGCNA) was performed to further screen the target genes. In addition, we used the receiver operating characteristic curve (ROC curve) to verify the diagnostic efficiency of target genes. Finally, target genes were validated by quantitative polymerase chain reaction (qPCR).

**Results:**

Four target genes (*CLUH, COG4, HADH*, and *MPZL2*) were discovered in early vascular endothelial impairment caused by T2DM through differential expression analysis and WGCNA. The ROC curve of target genes showed that *HADH* had the best diagnostic efficacy in VECD and AS. qPCR showed that the mRNA level expression of *HADH* and *MPZL2* were decreased in human coronary artery endothelial cells (HCAECs) treated with high glucose and palmitic acid.

**Conclusion:**

*HADH* may be the target gene in early VECD caused by T2DM.

## Introduction

With the improvement of living standards, the incidence of type 2 diabetes mellitus (T2DM) has an increasing tendency and its complication gradually received people's attention (1). Atherosclerosis (AS) is an important complication in T2DM (2). Patients with T2DM are more likely to have AS than those without T2DM for glucose and lipid metabolism disorders (3). In addition, T2DM speeds up the progression of AS with more inflammatory infiltration and necrotic area. Aortic AS, especially coronary AS, can cause poor blood flow and tissue ischemia, resulting in chest tightness, chest pain, and other clinical symptoms. Thus, it is crucial to recognize the molecular mechanism of AS caused by T2DM in the early stage.

**Figure 1:**
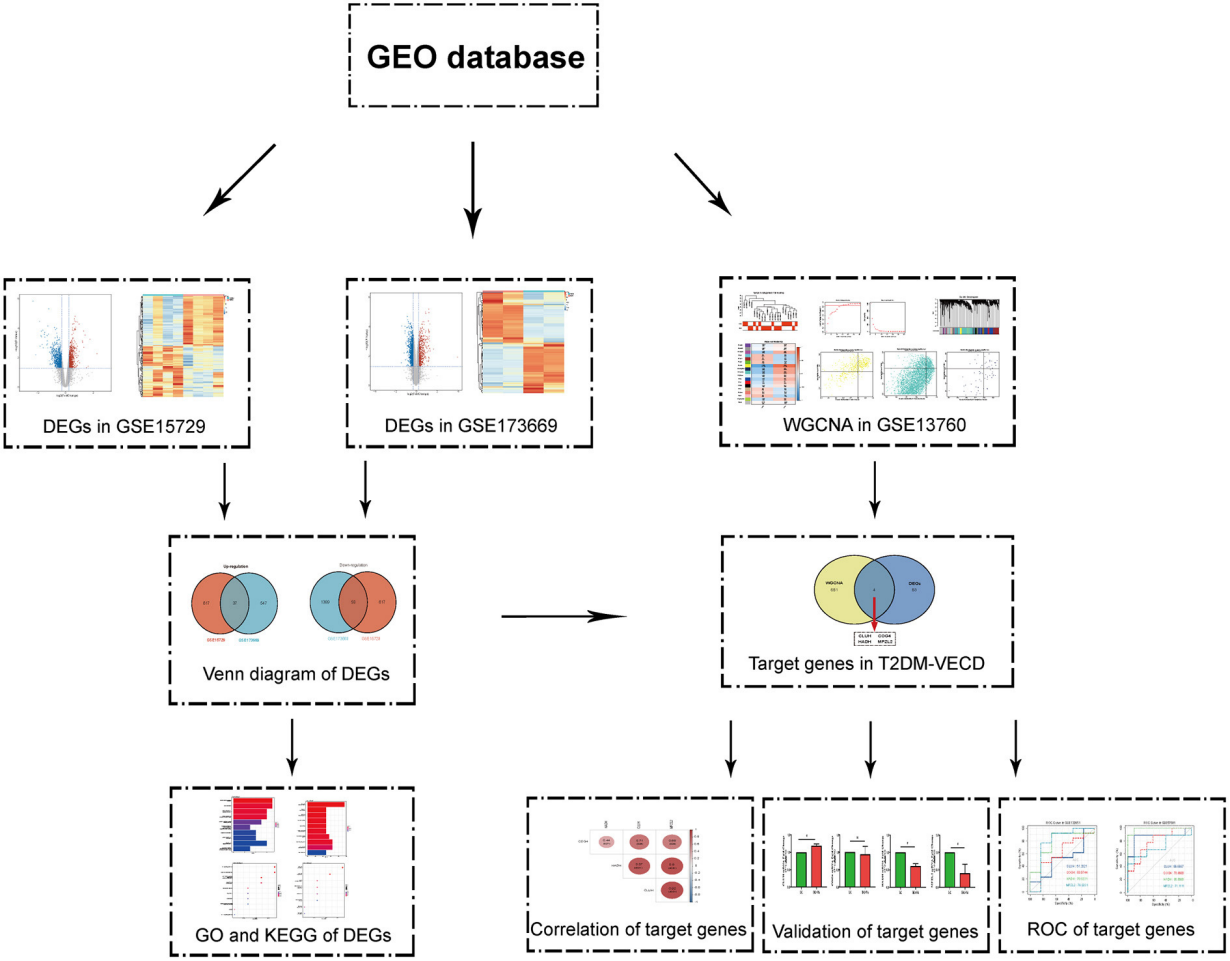
The flowchart of the study.

Endothelial cells, the regulators of vascular homeostasis, can regulate cell adhesion, angiogenesis, thrombogenesis, stability of blood flow, and so on (4). The occurrence of AS is closely associated with vascular endothelial cell dysfunction (VECD) (5). Researches show that inflammation increment, oxidative stress imbalance, the toxicity of glycolipids, platelet hyperactivity, and mitochondrial dysfunction may participate in VECD of T2DM (4, 6, 7). Programmed cell deaths, such as autophagy, apoptotic, and ferroptosis were also discovered in it (8–10). However, in spite of a lot of research on its mechanism, it is no effective target to be found to prevent AS of T2DM at present. Clinically, it seems that we can only avoid the incident of AS in T2DM patients by controlling blood glucose and lipids. Transcriptome sequencing analysis was greatly applied to identify the molecular mechanism of diseases with the development of sequencing technology (11, 12). Thus, we hope to uncover the underlying mechanism and screen the target molecule to prevent VECD from progressing to AS in T2DM *via* bioinformatics analysis. The flowchart of our study was summarized in Figure 1.

## Materials and methods

### Data acquisition

Through searching for vascular tissues or cells transcriptome datasets related to T2DM in Gene Expression Omnibus (GEO, https://www.ncbi.nlm.nih.gov/geo/), we only discovered three datasets related to early VECD of T2DM. They were GSE15729, GSE173669, and GSE13760. GSE15729, the study about aorta issues sequencing in type 2 diabetic AS model mice, contained 4 T2DM groups and 4 NDM (non-diabetes) groups (13). GSE173669, the study about human umbilical vein endothelial cells (HUVECs) sequencing stimulated by high glucose and ox-LDL *in vitro*, included 2 T2DM groups and 2 NDM groups (14). GSE13760 was comprised of 10 arterial tissues from type 2 diabetic patients and 11 arterial tissues from non-diabetic patients (15). We chose datasets GSE132651 about coronary endothelial dysfunction and GSE57691 about AS to validate the diagnostic efficiency of target genes. GSE132651 contained 6 normal groups and 13 coronary endothelial dysfunction groups, while GSE57691 contained 10 normal groups and 9 AS groups (16, 17).

### Selection of differential expression genes

Differential expression genes (DEGs) in datasets, GSE15729 and GSE173669, were screened by “limma” package in R respectively with *p*-value < 0.05 and |log2 fold change (FC)| >log2 (1.2). The results of DEGs were visualized as volcano plots and heatmap *via* R. The common up-regulated and down-regulated DEGs in two datasets were presented in the Venn diagram through the online tool Hiplot (https://hiplot.com.cn/).

### Functional enrichment analysis

Functional enrichment analysis, which includes gene ontology (GO) and Kyoto encyclopedia of genes and genomes (KEGG), was applied to assess the function, location, or pathway of DEGs. We employed the “clusterProfile” package in R to perform GO and KEGG analyses and exhibited the results as bar and bubble diagrams separately (18).

### Weighted gene co-expression network analysis

Weighted gene co-expression network analysis (WGCNA) is a great method to explore the connection between phenotypic character and genes (19). Considering that WGCNA requires a large sample size, we used dataset GSE13760 to perform WGCNA. Firstly, missing values and outliers were identified and outlier samples were deleted if there are outliers in GSE13760. The topological overlap matrix (TOM) was constructed by selecting the appropriate soft threshold β. Then, a hierarchical clustering tree of module identification was generated. Finally, the relationship between module and trait was calculated to pick out the first three modules with the strongest correlation, and module-trait genes were identified by using module membership (MM) >0.8 and gene significance (GS) >0.5 or 0.4 as the screening criteria.

### Identification of target genes in VECD of T2DM

The module-trait genes in WGCNA were intersected with DEGs to gain target genes associated with VECD of T2DM. Gene correlation analysis and receiver operating characteristic curve (ROC curve) were performed to discover the correlation and diagnostic value of the obtained target genes.

### Cell culture and quantitative polymerase chain reaction

Human coronary artery endothelial cells (HCAECs) and their complete medium were purchased from Procell Life Science and Technology (Wuhan, China). HCAECs were cultured at 37°C in an air saturation of 5% CO_2_. The cells were overgrown to 80–90% for passage. We used high glucose (HG, 33.3 mM) and palmitic acid (PA, 200 mM) to treat HCAECs for 24 h. Low glucose (LG, 5.5mM) without PA was as a control. Total RNA was extracted from HCAECs using Trizol reagent (Beyotime Biotechnology, Shanghai, China). The concentration of total RNA was measured and RNA was reverse-transcribed into cDNA by TranScript All-in-One First-Strand cDNA Synthesis SuperMix for the qPCR kit (Transgen, Beijing, China). The diluted cDNA was processed for qPCR with Top Green qPCR SuperMix kit (Transgen, Beijing, China). The primers of *CLUH, COG4, HADH*, and *MPZL2* were synthesized by Tsingke Biotechnology (Beijing, China). The sequences were as follows:

*CLUH*: forward 5′-GTGATGGAGTACGACCTGTC-3′
  reverse 5′-GAACTCAGCTTTGCTCTCGTAG-3′;
*COG4*: forward 5′-GATGCTCTTTTGGAACAGCAAA-3′
  reverse 5′-CTCTCTGAATGGCCTGATAGAG-3′;
*HADH*: forward 5′-GGCCAAGAAGATAATCGTCAAG-3′
  reverse 5′-TGGTCTACCAACACTACTGTG-3′;
*MPZL2*: forward 5′-ATGTATGGCAAGAGCTCTACTC-3′
  reverse 5′-TGGAGAAAGTGCATTTTAACCG-3′.

All experiments were repeated at least three times.

### Statistical analysis

GraphPad Prism 8.0 was used to perform statistical analysis and drawing. We selected a paired t-test as the statistical method. *p* < 0.05 was considered statistically significant. Data were presented as mean ± standard deviation (SD).

## Results

### Screening of differential expression genes

**Figure 2:**
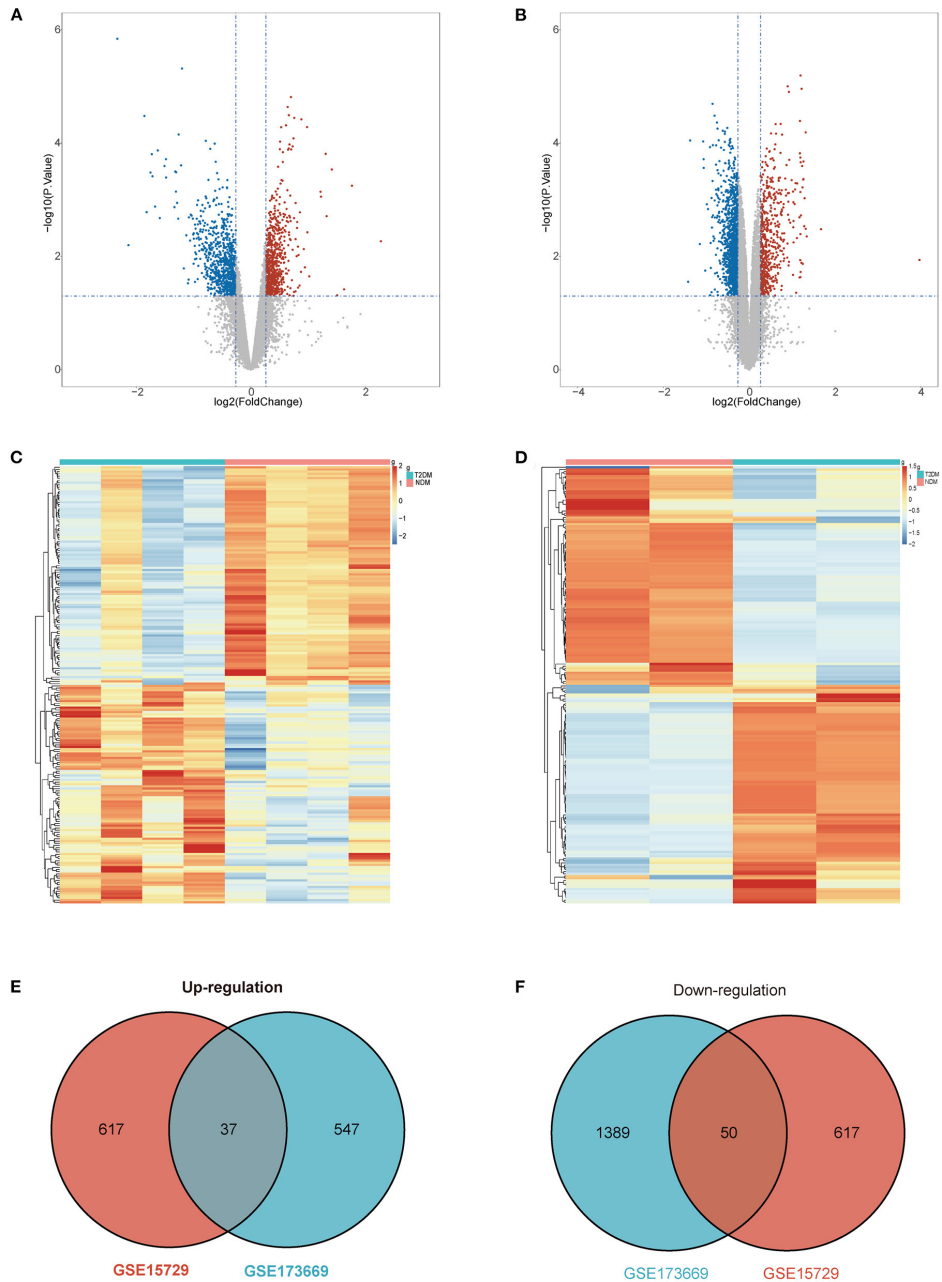
Differential expression genes (DEGs). **(A)** Volcano plot of GSE15729. **(B)** Volcano plot of GSE173669. **(C)** Heatmap of GSE15729. **(D)** Heatmap of GSE173669. **(E)** Venn diagram of common up-regulated DEGs from the two datasets. **(F)** Venn diagram of common down-regulated DEGs from the two[[Inline Image]] datasets.

Datasets GSE15729 and GSE173669 were downloaded from GEO and analyzed by “limma” package in R. We screened 654 up-regulated and 667 down-regulated DEGs in GSE15729. In the meantime, 584 up-regulated and 1,439 down-regulated DEGs were identified in GSE173669. The results of DEGs were visualized as volcano plots and the top 200 DEGs were shown in the heatmap (Figures 2A–D; Supplementary Table 1). The common DEGs with up-regulation and down-regulation in two datasets were obtained respectively and exhibited in Venn diagrams. A total of 87 common DEGs (37 up-regulated genes and 50 down-regulated genes) were selected eventually (Figures 2E,F).

### Functional enrichment analysis of DEGs in VECD of T2DM

**Figure 3:**
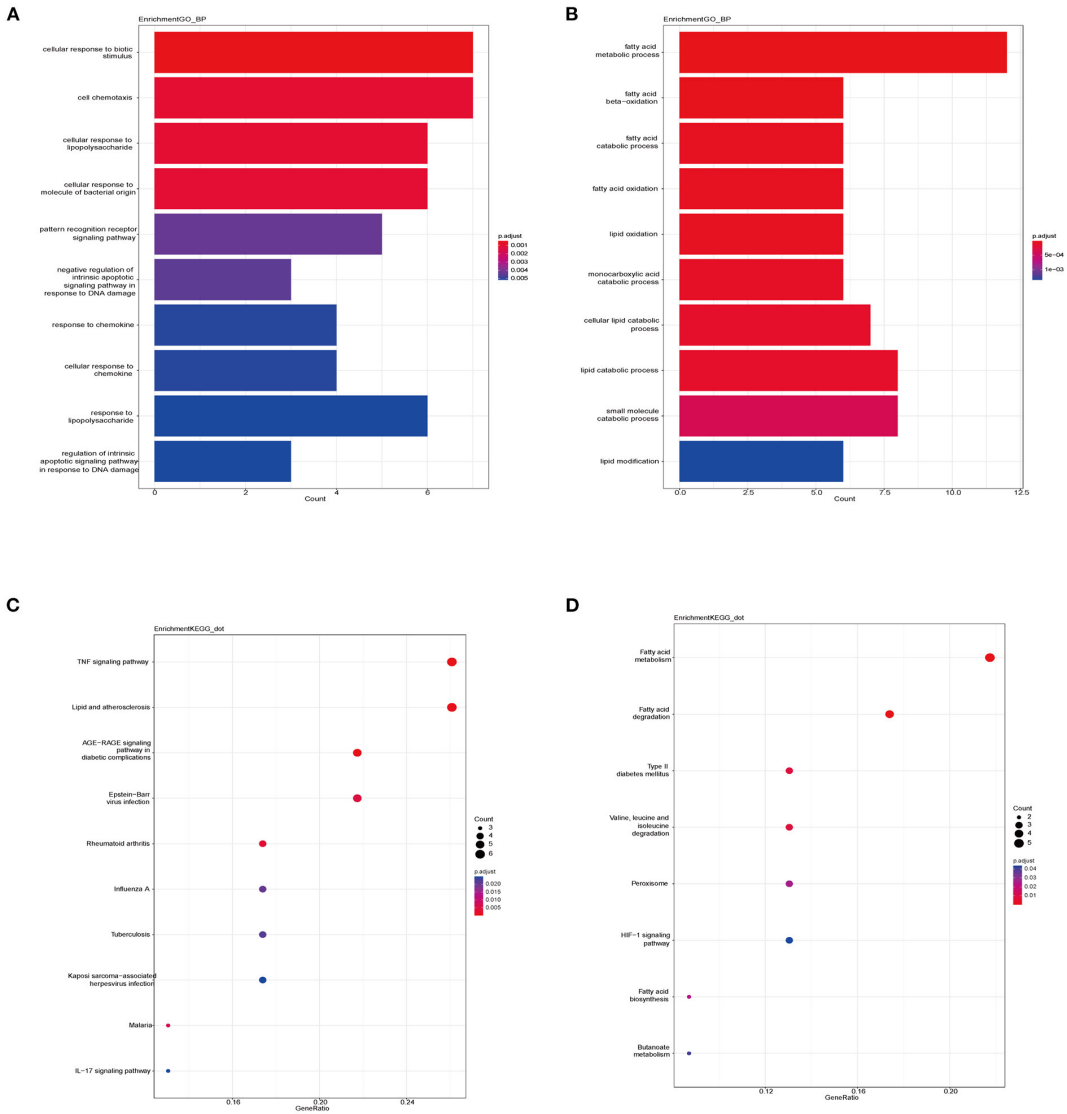
Functional enrichment of differential expression genes (DEGs). **(A)** Biological process (BP) of up-regulated DEGs in gene ontology (GO) analysis. **(B)** BP of down-regulated DEGs in GO analysis. **(C)** Pathways of up-regulated DEGs in Kyoto encyclopedia of genes and genomes (KEGG) analysis. **(D)** Pathways of down-regulated DEGs in KEGG analysis.

We applied the “clusterProfile” package in R to perform functional enrichment analysis, which contained GO and KEGG analyses (Supplementary Table 2). The biological process (BP) of up-regulated DEGs in VECD of T2DM was enriched in cellular response to biotic stimulus, cellular response to lipopolysaccharide, and cell chemotaxis (Figure 3A), while BP of down-regulated DEGs was enriched in the fatty acid metabolism process, fatty acid beta-oxidation and catabolic process (Figure 3B). According to KEGG analysis, up-regulated DEGs in VECD of T2DM participated in the TNF signaling pathway, lipid and AS, and Advanced glycation end product (AGE)-receptor for AGE (RAGE) (AGE-RAGE) signaling pathway in diabetic complications (Figure 3C). Down-regulated DEGs in VECD of T2DM were involved in fatty acid metabolism, fatty acid degradation, and type 2 diabetes mellitus (Figure 3D).

### Weighted gene co-expression network analysis and trait-module genes

**Figure 4:**
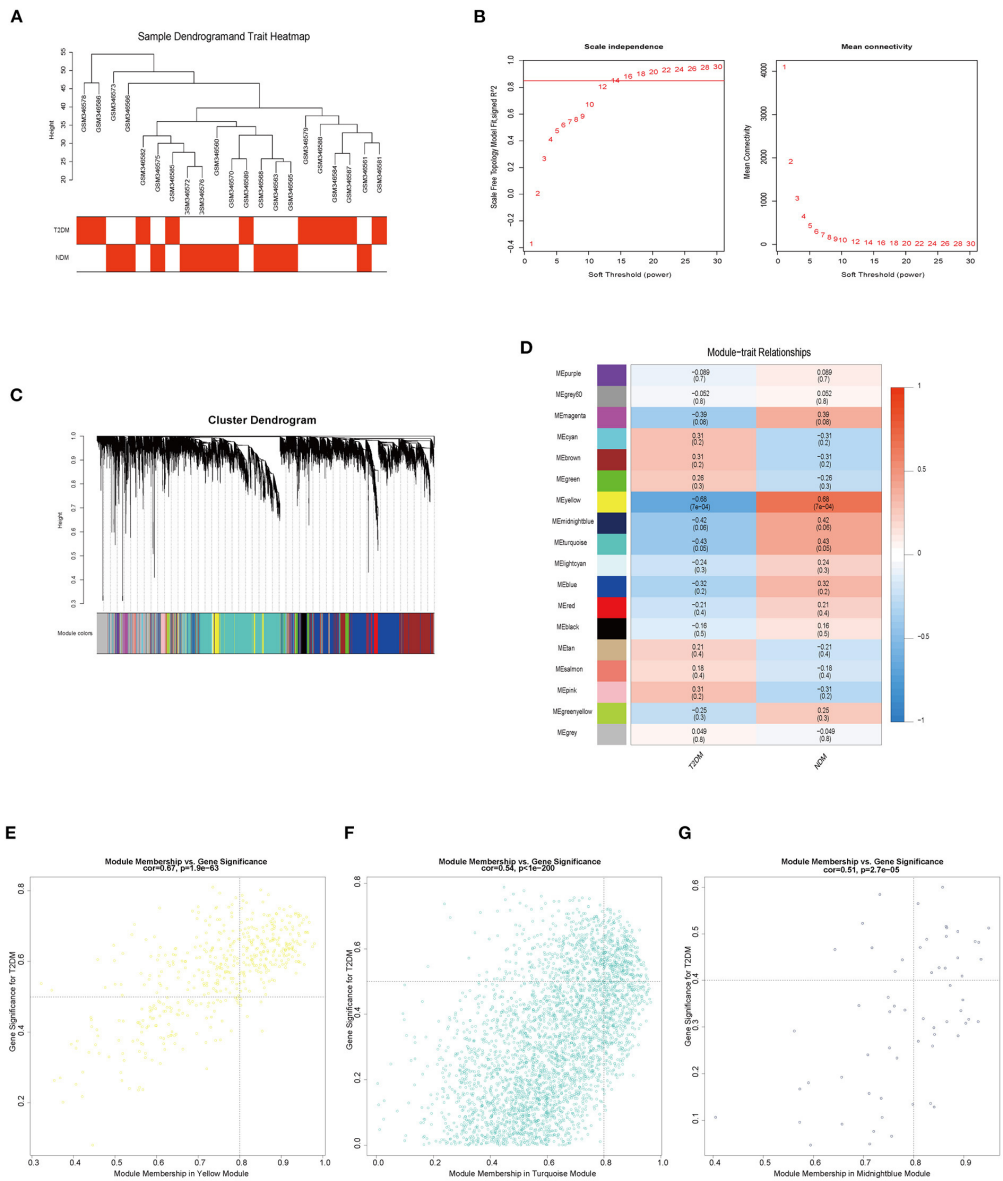
The results of weighted gene co-expression network analysis (WGCNA). **(A)** Sample clustering and trait heatmap of GSE13760. **(B)** The relationship of soft threshold and scale independence or mean connectivity. **(C)** Cluster dendrogram and partitioned modules. **(D)** The eigengene adjacency heatmap of the correlation between modules and vascular endothelial cell dysfunction (VECD) of type 2 diabetes mellitus (T2DM). **(E)** The scatter plot of the yellow module eigengenes. **(F)** The scatter plot of the turquoise module eigengenes. **(G)** The scatter plot of the midnight blue module eigengenes.

In order to further discern the target genes in VECD of T2DM, we used dataset GSE13760 to perform weighted gene co-expression network analysis (WGCNA). There was no outlier in sample clustering on the basis of the Pearson correlation coefficient (Figure 4A). We found the best soft threshold β to be 16 when a scale-free *R*^2^ is equal to 0.85 (Figure 4B). Based on β = 16, we constructed 18 gene co-expression modules *via* average hierarchical clustering and dynamic tree clipping (Figure 4C). The gray module represented genes not classified in other modules. In the correlation analysis of module and trait, yellow, turquoise, and midnight blue modules were the top three modules with the strongest correlation with VECD of T2DM (Figure 4D). According to set the screening criteria as MM > 0.8 as well as GS > 0.5 in yellow and turquoise modules and GS > 0.4 in midnight blue module, 685 trait-module genes were selected from three modules (Figures 4E–G; Supplementary Table 3).

### Identification of target genes in VECD of T2DM

**Figure 5:**
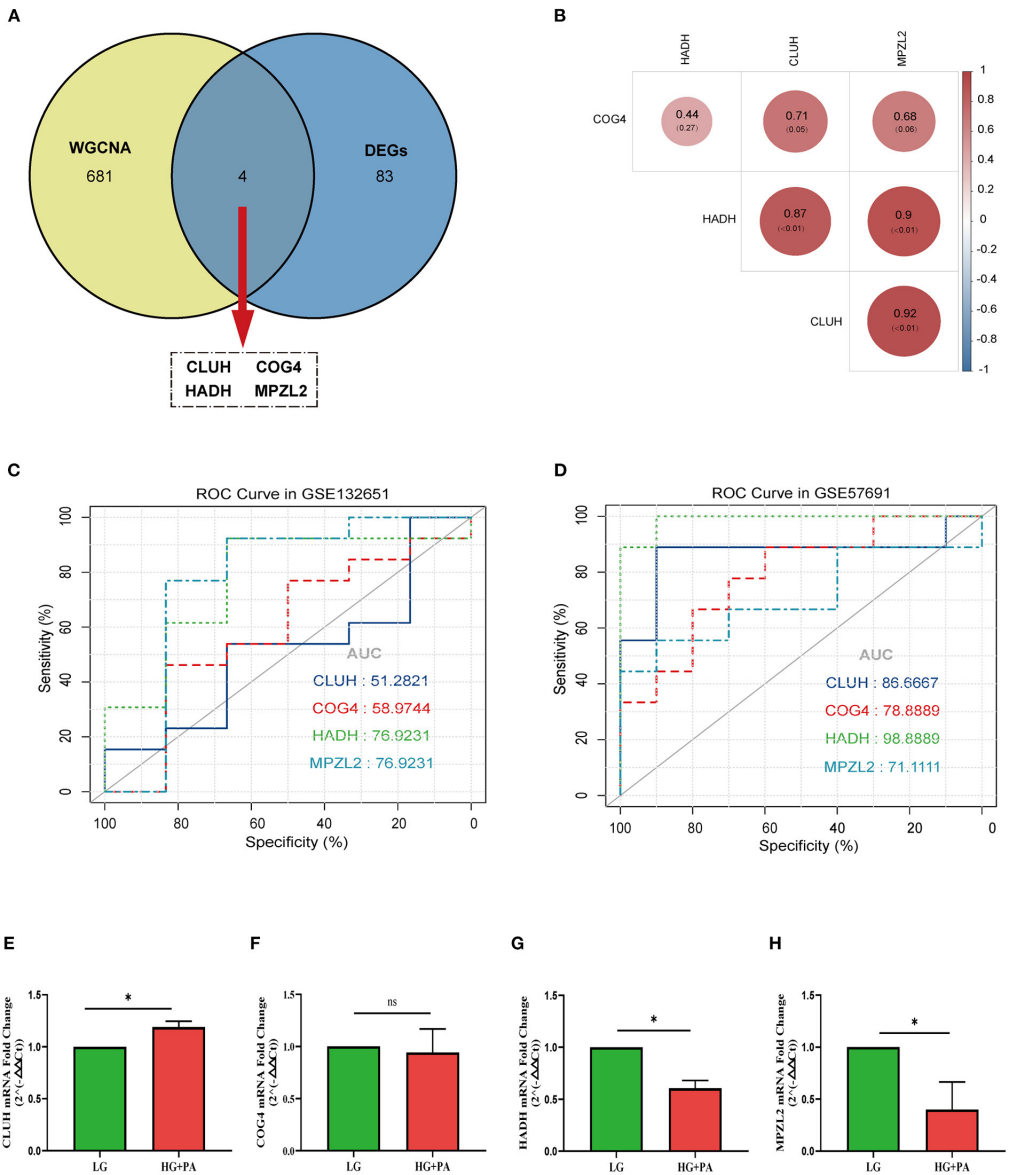
Analysis and validation of target genes. **(A)** Venn diagram of common genes between differential expression genes (DEGs) and trait-module genes in weighted gene co-expression network analysis (WGCNA). **(B)** The correlation analysis of target genes. **(C)** Receiver operating characteristic curve (ROC curve) of target genes in GSE132651. **(D)** ROC curve of target genes in GSE57691. The mRNA fold change of *CLUH* **(E)**, *COG4* **(F)**, *HADH* **(G)**, and *MPZL2* **(H)** in human coronary artery endothelial cells (HCAECs) treated with high glucose (HG) and palmitic acid (PA). * *p* < 0.05; ns *p* > 0.05.

Four target genes (*CLUH, COG4, HADH*, and *MPZL2*) were selected by taking the intersection of DEGs and trait-module genes (Figure 5A). The correlation of target genes was displayed in Figure 5B. There was a strong positive correlation between *CLUH* and *MPZL2*. In addition, considering that there was no suitable dataset about VECD of T2DM with clinical information, we chose dataset GSE132651 about coronary endothelial dysfunction and dataset GSE57691 about AS to validate the diagnostic efficiency of target genes. In the stage of coronary endothelial impairment (Early AS), areas under the curve (AUC) of *HADH* and *MPZL2* were the maximum (Figure 5C). Furthermore, *HADH* had a significantly diagnostic accuracy in AS (Figure 5D).

### Validation of target genes

We performed qPCR to verify the target genes in HCAECs treated with HG and PA *in vitro*. Expression of *HADH* and *MPZL2*, not *COG4* and *CLUH*, were significantly decreased (*p* < 0.05), which fit the results of transcriptome sequencing (Figure 5E).

## Discussion

Hyperglycemia, hyperlipidemia, and insulin resistance in T2DM are the crucial factors for the formation or acceleration of AS (4). Current studies suggested that VECD happened in the early process of AS and contributed to promoting the progress and complication of AS (20). Identification of VECD in T2DM will be beneficial for us to early diagnose and cure it as well as prevent the appearance of AS. However, there is no effective and convenient method to discover VECD in T2DM through extensive research about it. Therefore, we hoped to identify the target molecules of VECD in T2DM and provide the theoretical basis for clinical diagnosis and treatment by bioinformatics analysis.

In the study, we selected DEGs and performed a functional enrichment analysis of common DEGs in two datasets related to VECD in T2DM. DEGs of VECD in T2DM were primarily connected with the lipid metabolism process and related pathways, which indicated that T2DM damaged endothelial cells mainly by affecting lipid metabolism in the absence of obvious AS. In order to identify the target molecules, we used WGCNA and found four possible target genes (*CLUH, COG4, HADH*, and *MPZL2*).

*HADH*, the key enzyme in beta-oxidative of fatty acids, encodes for the enzyme 3-hydrocyacyl-coenzyme A dehydrogenase. The reduction of fatty acid beta-oxidation raises free fatty acids (FFAs). Plasma FFAs levels significantly increase in patients with T2DM, which is a crucial factor leading to VECD and AS (21). On the one hand, a high concentration of FFA causes mitochondrial respiratory chain electron transfer dysfunction and increases the generation of reactive oxygen species (ROS). On the other hand, FFA reacts with ROS to produce respiratory chain inhibitors and damage the biological oxidation process of mitochondria, resulting in the deposition of FFAs in mitochondria, forming a vicious cycle. In addition, high concentrations of FFAs impair antioxidant capacity by reducing the production of reduced glutathione (20). Combining with our study, increased FFAs levels may be related to the down-regulation of *HADH* caused by T2DM. Research shows that the siRNA knockdown of *HADH* influenced lipid storage and metabolism (22). Another research reveals that *HADH* expression is different in type 2 diabetic islet β cell dysfunction (23). In a meta-analysis of epigenome-wide association study (EWAS) of incident type 2 diabetes, genetic analysis of blood from patients collected 7–10 years before the diagnosis of T2DM showed significant enrichment in lipid metabolism (24). However, there is no relevant research on *HADH* and VECD of T2DM. Our study may provide the preliminary theoretical support for researching *HADH* on VECD of T2DM. *MPZL2* encodes myelin protein zero-like 2, an adhesion molecule that regulates epithelial cell interaction (25). There is very little research on *MPZL2* and only one study mentioned its protective effect on vascular endothelial cells (26). *HADH* and *MPZL2* were both decreased in VECD of T2DM and positively correlated. In addition, according to ROC analysis, the two were of equal diagnostic value in the early VECD. However, *HADH*, not *MPZL2*, has a significant diagnostic value in the AS stage, which indicates that *HADH* may play an important role in AS induced by sustained damage to endothelial cells caused by T2DM. Based on bioinformatics results and the important role of *HADH* in energy metabolism, we considered that *HADH* may be the target molecule in early vascular endothelial impairment in T2DM.

*CLUH*, a cytosolic protein, participates in mitochondrial biogenesis. It can regulate mitochondrial energy metabolism and adipogenesis (27, 28). *CLUH*-knockout cells show decreased abundance of respiratory complexes due to alterations in mitochondrial translation and conversion of glycolipid metabolism (28). These previous researches are consistent with the result of *CLUH* down-regulation in VECD of T2DM by bioinformatics analysis in this study. However, the qPCR result showed that the mRNA expression of *CLUH* was increased in HCAECs treated with HG and PA *in vitro*, which is contrary to the bioinformatics results. This may be related to the early protective increase of *CLUH* after coronary artery stimulation with high sugar and high fat.

Despite some heterogeneity, the study includes all available datasets about VECD of T2DM at present and performs bioinformatics analysis at the cell, animal, and human tissue levels. However, there are some limitations to our study. We did not analyze the diagnostic efficiency of the target gene in AS of T2DM or NDM sample for the lack of relevant data. In addition, the availability of the target gene still needs to be confirmed *in vivo*.

## Conclusion

In general, we attempted to ascertain the mechanism and target genes in VECD of T2DM by bioinformatics analysis of transcriptome sequencing. In particular, we found that the VCED in early AS caused by T2DM was mainly involved in the damage of lipid metabolism and *HADH* may be the target gene in it.

## Data availability statement

The original contributions presented in the study are included in the article/Supplementary material, further inquiries can be directed to the corresponding authors.

## Ethics statement

Ethical review and approval was not required for this study in accordance with the local legislation and institutional requirements.

## Author contributions

HY designed the research, searched data, and wrote a manuscript. HY and RW performed data processing and analysis. JW and YW participated in the discussion. XZ and LW revised the manuscript and confirmed the final draft with HY. All authors contributed to the article and approved the submitted version.

## Funding

This study was supported by the Talent Introduction Funding Project of the First Affiliated Hospital of Jinan University (No. 808026) and the Basic Scientific Research Project of Central Universities of Jinan University (No. 21622301).

## Conflict of interest

The authors declare that the research was conducted in the absence of any commercial or financial relationships that could be construed as a potential conflict of interest.

## Publisher's note

All claims expressed in this article are solely those of the authors and do not necessarily represent those of their affiliated organizations, or those of the publisher, the editors and the reviewers. Any product that may be evaluated in this article, or claim that may be made by its manufacturer, is not guaranteed or endorsed by the publisher.

## Abbreviations

T2DM: Type 2 diabetes mellitus
NDM: Non-diabetes
AS: Atherosclerosis
DEGs: Differential expression genes
WGCNA: Weighted gene co-expression network analysis
VECD: Vascular endothelial cell dysfunction
HCAECs: Human coronary artery endothelial cells
GO: Gene ontology
KEGG: Kyoto encyclopedia of genes and genomes
ROC: Receiver operating characteristic
AUC: Area under the curve
ROS: reactive oxygen species.

## References

[1] Zheng Y Ley SH Hu FB. Global aetiology and epidemiology of type 2 diabetes mellitus and its complications. Nat Rev Endocrinol. (2018) 14:88–98. 10.1038/nrendo.2017.151 29219149

[2] Einarson TR Acs A Ludwig C Panton UH. Prevalence of cardiovascular disease in type 2 diabetes: a systematic literature review of scientific evidence from across the world in 2007–2017. Cardiovasc Diabetol. (2018) 17:83. 10.1186/s12933-018-0728-6 29884191 PMC5994068

[3] Emerging Risk Factors C Sarwar N Gao P Seshasai SR Gobin R Kaptoge S. Diabetes mellitus, fasting blood glucose concentration, and risk of vascular disease: a collaborative meta-analysis of 102 prospective studies. Lancet. (2010) 375:2215–22. 10.1016/S0140-6736(10)60484-9 20609967 PMC2904878

[4] Kaur R Kaur M Singh J. Endothelial dysfunction and platelet hyperactivity in type 2 diabetes mellitus: molecular insights and therapeutic strategies. Cardiovasc Diabetol. (2018) 17:121. 10.1186/s12933-018-0763-3 30170601 PMC6117983

[5] Sitia S Tomasoni L Atzeni F Ambrosio G Cordiano C Catapano A. From endothelial dysfunction to atherosclerosis. Autoimmun Rev. (2010) 9:830–4. 10.1016/j.autrev.2010.07.016 20678595

[6] Yuan T Yang T Chen H Fu D Hu Y Wang J. New insights into oxidative stress and inflammation during diabetes mellitus-accelerated atherosclerosis. Redox Biol. (2019) 20:247–60. 10.1016/j.redox.2018.09.025 30384259 PMC6205410

[7] Vasquez-Trincado C Garcia-Carvajal I Pennanen C Parra V Hill JA Rothermel BA. Mitochondrial dynamics, mitophagy and cardiovascular disease. J Physiol. (2016) 594:509–25. 10.1113/JP271301 26537557 PMC5341713

[8] Tian J Popal MS Liu Y Gao R Lyu S Chen K. Ginkgo biloba leaf extract attenuates atherosclerosis in streptozotocin-induced diabetic ApoE–/– mice by inhibiting endoplasmic reticulum stress via restoration of autophagy through the mTOR signaling pathway. Oxid Med Cell Longev. (2019) 2019:8134678. 10.1155/2019/8134678 31080547 PMC6442448

[9] Jin ZH Gao P Liu ZT Jin B Song GY Xiang TY. Composition of ophiopogon polysaccharide, notoginseng total saponins and rhizoma coptidis alkaloids inhibits the myocardial apoptosis on diabetic atherosclerosis rabbit. Chin J Integr Med. (2020) 26:353–60. 10.1007/s11655-018-3014-2 30328567

[10] Meng Z Liang H Zhao J Gao J Liu C Ma X. HMOX1 upregulation promotes ferroptosis in diabetic atherosclerosis. Life Sci. (2021) 284:119935. 10.1016/j.lfs.2021.119935 34508760

[11] Kalayinia S Goodarzynejad H Maleki M Mahdieh N. Next generation sequencing applications for cardiovascular disease. Ann Med. (2018) 50:91–109. 10.1080/07853890.2017.1392595 29027470

[12] Zhong M Wu Y Ou W Huang L Yang L. Identification of key genes involved in type 2 diabetic islet dysfunction: a bioinformatics study. Biosci Rep. (2019) 39:BSR20182172. 10.1042/BSR20182172 31088900 PMC6542763

[13] Bu DX Rai V Shen X Rosario R Lu Y D'Agati V. Activation of the ROCK1 branch of the transforming growth factor-beta pathway contributes to RAGE-dependent acceleration of atherosclerosis in diabetic ApoE-null mice. Circ Res. (2010) 106:1040–51. 10.1161/CIRCRESAHA.109.201103 20133903 PMC2848909

[14] Chao ML Luo S Zhang C Zhou X Zhou M Wang J. S-nitrosylation-mediated coupling of G-protein alpha-2 with CXCR5 induces Hippo/YAP-dependent diabetes-accelerated atherosclerosis. Nat Commun. (2021) 12:4452. 10.1038/s41467-021-24736-y 34294713 PMC8298471

[15] Skov V Knudsen S Olesen M Hansen ML Rasmussen LM. Global gene expression profiling displays a network of dysregulated genes in non-atherosclerotic arterial tissue from patients with type 2 diabetes. Cardiovasc Diabetol. (2012) 11:15. 10.1186/1475-2840-11-15 22340758 PMC3348024

[16] Hebbel RP Wei P Milbauer L Corban MT Solovey A Kiley J. Abnormal endothelial gene expression associated with early coronary atherosclerosis. J Am Heart Assoc. (2020) 9:e016134. 10.1161/JAHA.120.016134 32673514 PMC7660702

[17] Biros E Gabel G Moran CS Schreurs C Lindeman JH Walker PJ. Differential gene expression in human abdominal aortic aneurysm and aortic occlusive disease. Oncotarget. (2015) 6:12984–96. 10.18632/oncotarget.3848 25944698 PMC4536993

[18] Yu G Wang LG Han Y He QY. clusterProfiler: an R package for comparing biological themes among gene clusters. OMICS. (2012) 16:284–7. 10.1089/omi.2011.0118 22455463 PMC3339379

[19] Langfelder P Horvath S. WGCNA: an R package for weighted correlation network analysis. BMC Bioinform. (2008) 9:559. 10.1186/1471-2105-9-559 19114008 PMC2631488

[20] Gimbrone MA Garcia-Cardena G. Endothelial cell dysfunction and the pathobiology of atherosclerosis. Circ Res. (2016) 118:620–36. 10.1161/CIRCRESAHA.115.306301 26892962 PMC4762052

[21] Ghosh A Gao L Thakur A Siu PM Lai CWK. Role of free fatty acids in endothelial dysfunction. J Biomed Sci. (2017) 24:50. 10.1186/s12929-017-0357-5 28750629 PMC5530532

[22] Kerr AG Sinha I Dadvar S Arner P Dahlman I. Epigenetic regulation of diabetogenic adipose morphology. Mol Metab. (2019) 25:159–67. 10.1016/j.molmet.2019.04.009 31031182 PMC6600120

[23] Lawlor N George J Bolisetty M Kursawe R Sun L Sivakamasundari V. Single-cell transcriptomes identify human islet cell signatures and reveal cell-type-specific expression changes in type 2 diabetes. Genome Res. (2017) 27:208–22. 10.1101/gr.212720.116 27864352 PMC5287227

[24] Fraszczyk E Spijkerman AMW Zhang Y Brandmaier S Day FR Zhou L. Epigenome-wide association study of incident type 2 diabetes: a meta-analysis of five prospective European cohorts. Diabetologia. (2022) 65:763–76. 10.1007/s00125-022-05652-2 35169870 PMC8960572

[25] Wesdorp M Murillo-Cuesta S Peters T Celaya AM Oonk A Schraders M. MPZL2, encoding the epithelial junctional protein myelin protein zero-like 2, is essential for hearing in man and mouse. Am J Hum Genet. (2018) 103:74–88. 10.1016/j.ajhg.2018.05.011 29961571 PMC6037131

[26] Miao Y Cui L Chen Z Zhang L. Gene expression profiling of DMU-212-induced apoptosis and anti-angiogenesis in vascular endothelial cells. Pharm Biol. (2016) 54:660–6. 10.3109/13880209.2015.1071414 26428916

[27] Cho E Jung W Joo HY Park ER Kim MY Kim SB. Cluh plays a pivotal role during adipogenesis by regulating the activity of mitochondria. Sci Rep. (2019) 9:6820. 10.1038/s41598-019-43410-4 31048716 PMC6497719

[28] Wakim J Goudenege D Perrot R Gueguen N Desquiret-Dumas V Chao JM. CLUH couples mitochondrial distribution to the energetic and metabolic status. J Cell Sci. (2017) 130:1940–51. 10.1242/jcs.201616 28424233

